# Ocular Trauma Score: case study and quiz

**Published:** 2015

A 65-year-old man suffered an injury to the right eye, caused by a stone which ricocheted while using a weed cutter in his garden at home. He had not been wearing eye protection. At initial assessment when he presented to the hospital 17 hours following the injury, his visual acuity was nil perception of light (NPL). He had a corneal perforation and early signs of endophthalmitis, including mucopurulent discharge and anterior uveitis, were already present. A CTscan showed no intraocular foreign body. Answer the questions and then compare them with how the team approached the situation. See article on page 44.

1. What is the raw score?
2. What is the ocular trauma score (OTS)?
3. What would you say to the patient and his family?
4. How would you treat the patient?
5. What is the likely clinical and visual outcome if the infection cannot be controlled?

Case study courtesy of Désirée C Murray

## ANSWERS

1. Raw score = 60 −17 (endophthalmitis), −14 (perforating Injury) = 29
2. OTS = 1
3. According to Table 2 (page 44), an OTS of 1 (the poorest prognosis) gives a probability of 73% that the final visual outcome will remain nil perception of light (NPL), and just a 17% probability that the patient would have perception of light (PL) at follow-up. From a clinical perspective, the presence of endophthalmitis, and VA of NPL at presentation, supported this finding and moreover suggests that the probability of regaining any vision is closer to 0%. The patient and their relatives had to be counselled with sensitivity (see page 50) about the prognosis.
4. In this instance, the team repaired the corneal full-thickness laceration and treated the patient with antibiotics (intravitreal vancomycin and ceftazidime; topical and oral moxifloxacin) and antifungals (intravitreal and topical amphotericin and topical natamycin).
5. Despite the above treatment, the infection failed to be controlled and the patient developed a corneal abscess. Having understood the extremely low probability of visual recovery and the risk of sympathetic ophthalmitis, the patient and his family accepted the need for removal of the eye and gave informed consent for the procedure. Evisceration of the ocular contents was performed 9 days after the injury.

## REFLECTIVE LEARNING

Visit **www.cehjournal.org** to complete the online ‘Time to reflect’ section.

